# High Temperature In Situ Optical Observation and Structural Optimization Numerical Simulation of High Nitrogen Steel (Cr18Mn18N)

**DOI:** 10.3390/ma15051805

**Published:** 2022-02-28

**Authors:** Shilong Zhu, Yi Kong, Wen Yue, Yunlong Tang

**Affiliations:** 1 School of Engineering and Technology, China University of Geosciences, Beijing 100083, China; 1002193107@cugb.edu.cn (S.Z.); 2102190009@cugb.edu.cn (Y.K.); yw@cugb.edu.cn (W.Y.); 2 Zhengzhou Institute, China University of Geosciences (Beijing), Zhengzhou 451283, China

**Keywords:** Cr18Mn18N, numerical simulation, surface structure, splicing

## Abstract

As a steel with high strength, good plasticity and fracture toughness, high temperature resistance, and corrosion resistance, Cr18Mn18N is widely used in industrial engineering and military fields. However, in a high temperature environment, Cr18Mn18N needs to be subjected to higher temperature, resulting in excessive expansion deformation and larger stress, which will greatly damage the stability and service life of the material structure. In this paper, the high temperature arc wind tunnel is used to heat the high nitrogen steel material with prefabricated round structure, and the surface images of the material are collected at the temperature of 1500 K. After comparison, it is found that the material is well preserved in a high temperature environment, indicating that the circular structure has better thermal protection ability. Based on the experiment, the thermal-fluid-solid coupling model is established, and the surface temperature field, deformation field, and stress field are analyzed. Different surface structures are designed, and numerical models of horizontal and vertical splicing components are established. Through numerical simulation, the surface structure is optimized, the surface temperature of the material is reduced, and the gap change trend of the splicing component is displayed. This work has theoretical significance for the application of materials in a high temperature environment and the optimization and improvement of material surface structure.

## 1. Introduction

With the development and growth of the aerospace, military, and national defense fields, extreme environments for material applications continue to emerge in various fields, such as hypersonic vehicles, manned spacecraft, and rockets, which will be subjected to extreme high temperature environments. Therefore, stricter application requirements are put forward for thermal protection materials. As a material applicated in extreme high temperature environments, it needs to have excellent properties, such as high temperature resistance, ablation resistance, and oxidation resistance, etc. In recent years, there has been more and more research on high temperature resistant materials, such as high temperature structural ceramics [1,2,3], high nitrogen steel [4,5], etc. In aerospace, aircraft, and other fields, steel, as an indispensable material, needs to withstand high temperatures in service. With the continuous use of steel, the research on the characteristics of steel under high temperature environments is gradually rising. For example, Wang et al. studied the mechanical properties of high-strength Q960 steel at high temperature through experiments, and a series of tensile coupon tests on Q960 steel was carried out under various temperatures from 20 to 900 °C. A new prediction equation for evaluating the properties of Q960 steel at high temperature was proposed [6]. The mechanical properties of structural steel at high temperature are very important for the fire-resistant design of steel structures. Chen et al. studied the mechanical properties of high-strength structural steel and low-carbon structural steel in a high-temperature environment. Through the high-temperature mechanical properties experiments of high-strength steel plate and low-carbon steel, their elastic modulus and yield strength at different levels were obtained. The tensile length and ultimate strength at high temperature were evaluated [7].

As a new type of steel, high nitrogen steel has the advantages of high strength, plasticity, good fracture toughness, high temperature resistance, and corrosion resistance. Because of its excellent performance in terms of high temperature resistance, it has gradually entered the field of high temperature applications. Matsubara et al. investigated the high temperature oxidation behavior of high nitrogen steel with about 0.3 mass% of nitrogen and compared it with nitrogen-free steels and ASME grade 91. High temperature oxidation tests were carried out in air over the temperature range 750–850 °C for 300 h at maximum. The results show that high nitrogen (0.3 mass% of nitrogen) promotes the formation of the oxide scale while a high concentration of Cr inhibits the oxidation process of high nitrogen steel. The experimental results show that the high nitrogen steel is well preserved at 750–850 °C and can withstand high temperature to be applied in high temperature environments [8]. Zhang et al. studied the relationship between nitrogen content, temperature, and pressure by preparing 18Cr18MnN austenitic stainless steel with nitrogen content greater than 0.9%. The result show that the nitrogen content in steel increases with increasing melting pressure, at air pressure 1.0 MPa < P < 1.4 MPa, the increase of nitrogen content in steel is evident particularly. In the 1813 K < T < 1913 K temperature range, the nitrogen content in steel decreases with the increasing temperature. There are differences in variation in the temperature range. The best thermodynamic conditions for smelting 18Mn18Cr austenitic stainless steel with a nitrogen content of 0.9% are: pressure and temperature of 1.32 MPa and 1873 K, respectively [9]. Ran et al. used scanning electronic microscopy and Raman spectroscopy to study the high temperature oxidation of duplex stainless steel (DSSs) 19Cr–10Mn–0.3Ni–xN (x = 0.211–0.315 wt.%). The ambient temperature was set at 1100 °C. The result show that DSSs with lower nitrogen content have poor high-temperature oxidation resistance. In the initial oxidation stage, a local breakaway region of the ferrite diffusion layer is produced. With the extension of the oxidation time, these areas can be repaired automatically. However, in DSSs with high nitrogen content, the high temperature oxidation phenomenon is obvious and penetrates the local breakaway regions as the increased outward diffusion of nitrogen ions [10].

As an important analysis tool, numerical simulation has the characteristics of low cost and high efficiency, and is widely accepted by research scholars [11,12]. For example, Ma et al. [13] studied the hot forming properties of the advanced high-strength steel B1500HS. The punch force–displacement curve is studied by combining experiment and numerical simulation, and the temperature distribution and temperature path of the sheet metal at the maximum punch load. The result show that the experimental and numerical simulation results maintain good consistency, which proves the predictive capability of finite element simulation for hot stamping. Sui et al. [14] established a numerical model of SA508-3 steel, studied the effect of temperature on steel, and observed the strain and strain rate on the microstructure. The results of experimental and numerical simulation demonstrated that temperature is an important factor in initiating dynamic recrystallization. Higher temperature means lower critical strain, so it can promote recrystallization to obtain a uniform fine structure.

In this paper, a high temperature arc wind tunnel test is carried out on high nitrogen steel. By collecting the image of the surface morphology of the material in the high temperature wind tunnel heating environment, it can be seen that the surface of the material is well preserved in the high temperature environment, and the circular array structure has not been damaged. This shows that the prefabricated circular array plays a good role in thermal protection of the material structure under a high temperature environment. At the same time, a thermal-fluid-solid coupling model was established by numerical simulation methods, and different surface structures and splices designed. Numerical analysis results show that the presence of the prefabricated structure reduces the surface temperature of the material and obtains the deformation field and stress distribution of the sample. Through the high temperature wind tunnel, the prefabricated hole material is heated and combined with numerical simulation to study the influence of prefabricated holes on the surface temperature and deformation of the material. The purpose is to improve the service life of high nitrogen steel in high temperature environment and provide theoretical and experimental support for the application of high nitrogen steel in extreme high temperature environments.

### 1.1. Theory

In this work, the change of material surface in a high temperature environment is obtained by in situ optical visualization technology. However, due to the complex environment and high cost of the high temperature arc wind tunnel experiment, it is difficult to carry out repeated experimental research on materials. Therefore, the temperature field, deformation field, and stress field of the material surface were analyzed by numerical simulation on the basis of experimental observation. Different surface structures and splicing forms were designed to optimize the material surface in a high temperature environment. The following is a brief introduction to the principles involved in the simulation.

#### 1.1.1. Governing Equations

The governing equations are as follows [15,16]:

Mass conservation equation:

(1) $$\frac{\partial \rho }{\partial t}+\nabla \cdot \left(pv\right)=0$$

Momentum conservation equation:

(2) $$\frac{\partial }{\partial t}\left(p\vec{v}\right)+\nabla \cdot \;\left(p\vec{v}\;\vec{v}\right)=-\nabla p+\nabla \cdot \left(\overset{=}{\tau }\right)+\rho \vec{g}+\vec{F}$$

Energy conservation equation:

(3) ∂(ρE)/∂t+∇·[(ρE+p)V]=ρF·V+∇·(k∇T) 

where *p* is the static pressure, ρ is the density,$\;\rho \vec{g}$ is the gravity, *F* is volume force, $\vec{v}$ is the fluid velocity with *x*, *y*, and *z* components, *E* is energy, *T* is temperature, and *k* is the effective thermal conductivity.

#### 1.1.2. Fluid-Solid Heat Transfer Equations

When solving the heat-fluid-solid coupling model, the calculation of the transient temperature field of the fluid can be regarded as a steady-state flow field [17] (the momentum equation and governing equation in the flow field can be ignored).

(4) ∇·(ρVϕ)=∇·(ΓVϕ)+S

where ρ is the density and *ϕ* is a variable that includes velocity components *U*, *V*, and *W* as well as temperature *T* in the *X*, *Y*, and *Z* directions. *Γ* is the generalised diffusion coefficient of each variable, and S is the source term of each variable.

In the solid model, energy transfer is defined as follows:

(5) $$\frac{\partial \left(\rho h\right)}{\partial t}=\nabla \cdot \left(k\nabla T\right)+S_{h}$$

where *h* is the sensible enthalpy and *S_h_* is the volumetric heat source. The right side of Equation (5) represents the heat flux generated by heat conduction and heat source. The left side represents the change of energy with time in the solid domain.

#### 1.1.3. Species Transport Equations

The species transport model is used to define the components and calculate the component migration, and the conservation equation is as follows [18]:

(6) $$\frac{\partial }{\;\partial t}\left(\rho Y_{i}\right)+\nabla \left(\rho \vec{v}Y_{i}\right)=-\nabla \vec{J_{l}}+R_{i}+S_{i}$$

where ρ is the density, $\vec{v}$ is the fluid velocity with *x*, *y*, and *z* components, *Y_i_* is mass fraction of each species, *R_i_* is the net rate of species *i* produced by chemical reactions, $\vec{J_{i}}$ is the diffusion flux term of species *i*, which is caused by the concentration and temperature gradient, and *S_i_* is the additional rate generated by discrete phases and user-defined terms.

#### 1.1.4. Stress-Strain Relationship

In this paper, the thermal expansion of the material model is analyzed. In the simulation analysis, the material is considered as linear elasticity, and the coefficient of thermal expansion is considered as a constant. Therefore, the stress–strain relationship of the material is as follows [19]:

(7) $$\varepsilon_{x}=\alpha_{x}∆T+\frac{\sigma_{x}}{E_{x}}-\frac{v_{\mathrm{xy}}\sigma_{y}}{E_{x}}-\frac{v_{\mathrm{xz}}\sigma_{z}}{E_{x}}\;\varepsilon_{y}=\alpha_{y}∆T-\frac{v_{\mathrm{xy}}\sigma_{x}}{E_{x}}+\frac{\sigma_{y}}{E_{y}}-\frac{v_{\mathrm{yz}}\sigma_{z}}{E_{y}}\;\varepsilon_{z}=\alpha_{z}∆T-\frac{v_{\mathrm{xy}}\sigma_{x}}{E_{x}}-\frac{v_{\mathrm{yz}}\sigma_{y}}{E_{y}}+\frac{\sigma_{z}}{E_{z}}\;\varepsilon_{\mathrm{xy}}=\frac{\sigma_{\mathrm{xy}}}{G_{\mathrm{xy}}}\;\varepsilon_{\mathrm{yz}}=\frac{\sigma_{\mathrm{yz}}}{G_{\mathrm{yz}}}\;\varepsilon_{\mathrm{xz}}=\frac{\sigma_{\mathrm{xz}}}{G_{\mathrm{xz}}}$$

where *E*, *v*, and *G* refer to Young’s modulus, Poisson’s ratio, and shear modulus, respectively. *Ε*_x_ and *σ_x_* is the normal strain and normal stress in the *x*-axis direction, and *ε_xy_* and *σ_xy_* are the shear strain and shear stress in the *xy* plane.

## 2. Experimental

### 2.1. Experimental Setup

**Figure 1:**
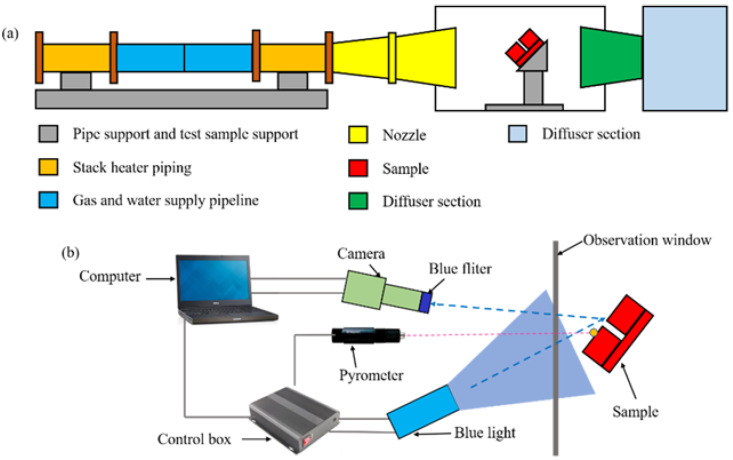
Schematic of the experimental equipment: (**a**) High-temperature wind tunnel; (**b**) data acquisition device.

In this paper, a high temperature wind tunnel is used to heat the materials in the test chamber [20,21]. The heating principle of high temperature arc wind tunnel is shown in Figure 1a. In the experiment, the working principle is as follows: At the beginning of the operation, the high-pressure airflow is heated by the stack heater and transported using a gas and water supply pipeline. Then, the gases expand and accelerate through the nozzle to form a high temperature jet that heats the specimen installed in the experimental chamber. After the experiment, airflow is allowed into the pipeline of the diffuser section, where it decelerates and then enters the vacuum vessel after cooling. In the experiment, the Mach number is about 1.7. The total enthalpy and maximum heat flux are within the range of 9000–15,000 MJ/kg and 2.0–2.5 MW/m^2^, respectively. In the high-temperature arc wind tunnel, the surface parameters of the specimen cannot be obtained by conventional optical observation techniques owing to high-temperature radiation, airflow interference, and other factors. Therefore, in order to obtain the behavior change of the material surface, the temperature synchronous deformation measurement device is used during experiment as shown in Figure 1b [22,23]. The equipment consists of a single-point infrared pyrometry (Raytek, MI3, Santa Cruz, CA, USA), a blue light source (central wavelength 465 nm), a CCD camera (SP-500-USB, JAI Ltd., Yokohama, Japan), a 75 mm lens (JAI Ltd.), and a blue bandpass filter (with central wavelength of 465 nm and full width at half maximum of 5 nm). The camera and blue light source were fixed at the observation windows outside the laboratory for image acquisition and light supplement.

The working principle of synchronous measurement is as follows: Firstly, the blue light source is used to compensate for the light on the sample surface. Secondly, the blue light source is reflected from the sample surface to the CCD camera, and the camera has a built-in blue band-pass filter to reduce the high temperature radiation and capture the change of material surface morphology. Finally, the digital image correlation method is used to analyze the deformation of the sample surface [24,25]. The single point infrared thermometer is used to obtain the temperature of the reference point, and then the improved two-color temperature measurement method is used to calculate the temperature field of the material surface [26,27].

(8) $$\ln\left(B_{RG}\right)-\ln\left(B_{RG0}\right)=C_{2}\left(\frac{1}{\lambda_{G}}-\frac{1}{\lambda_{R}}\right)\left(\frac{1}{T}-\frac{1}{T_{0}}\right)$$

where *T*_0_ is the temperature of the reference point, which is measured using a single-point infrared thermometer, *C*_2_ is the optical constant, and *B_RG_* is the ratio of the intensity of the red to green light at the measuring point. *λ_G_* and *λ_R_* are the wavelengths of the red and green light, respectively.

### 2.2. Material Selection and Preparation

The Mn18Cr18N has excellent corrosion resistance and high temperature oxidation resistance, which is the basis of long-term service in extreme environments. The test material used was an Mn18Cr18N steel ingot. The preparation process began with electric furnace refining. Secondly, the ingot was cast under the protection of nitrogen, and the electroslag remelting electrode is forged. Finally, electroslag was remelted and cast into steel ingots. The test material was taken from the nozzle end of the electroslag remelting steel ingot in order to reduce the surface temperature of the surface material, further extending its service life. Therefore, laser drilling equipment is used to prepare pores on the material surface, and inert gas is introduced into the pores to reduce the surface temperature of the material in the high-temperature environment and thermal shock environment and protect the material at the same time.

### 2.3. Experimental Results

**Figure 2:**
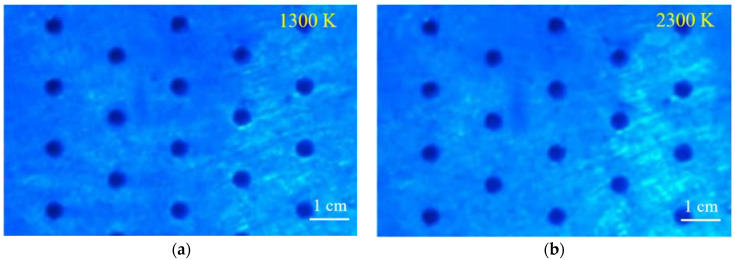
Comparison of surface images of high nitrogen steel materials with pores before and after high temperature heating: (**a**) before heating up; (**b**) after heating up.

In the high-temperature arc wind tunnel experiment, in-situ optical visualization technology was used to observe the surface of the material. The experimental results are shown in Figure 2. Figure 2a was collected at the initial stage of heating. The surface temperature of the material is about 1300 K, and an array of circular holes is prefabricated on the surface of the material. Figure 2b was collected in the later stage of high-temperature arc wind tunnel heating. The surface temperature of the material is approximately 1500 K. By comparing two images at different times, it can be seen that the material surface is well preserved in the high temperature environment of 1500 K, which indicates that the prefabricated circular array has a better thermal protection effect on the material. The blue color of the two pictures is due to the use of a blue light source to fill the light on the surface of the material.

**Figure 3:**
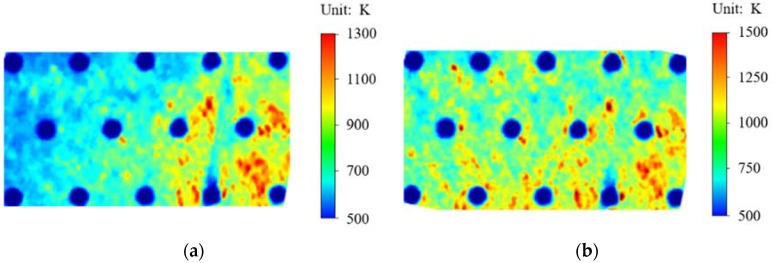
Nephogram of surface temperature of high nitrogen steel with pores before and after heating: (**a**) before heating up; (**b**) after heating up.

Figure 3 shows the temperature nephogram before and after the heating up. Figure 3a is the temperature nephogram before heating up, and Figure 3b is the temperature nephogram after heating up. It can be seen from the comparison that due to the existence of the surface circular array structure, the surface temperature of the material is reduced under high temperature heating, and the local surface temperature rise of the material is low. The material and its structure are well preserved. The results show that the circular array structure has a good decreasing temperature effect and a good thermal protection effect on the material.

Because the characteristic points of the material surface change greatly under the scouring and ablation of the high-temperature wind tunnel environment, and DIC uses the characteristic points for displacement calculation, the ellipse fitting method can accurately show the displacement of the material in the calculation of displacement field. The ellipse fitting method based on least square method is used to fit it, and the displacement change of pores is displayed. The ellipse fitting based on the least square method is an optimal estimation technique introduced by the maximum likelihood method when the random error is a normal distribution, which can minimize the sum of squares of the measurement error. By looking for a set of parameters, the distance metric between the data point and the ellipse is minimized. Minimize the distance metric between the data point and the ellipse by looking for a set of parameters. The method is as follows:

The general elliptic equation is:

(9) $$Ax^{2}+Bxy+Cy^{2}+Dx+Ey+F=0$$

where *A*, *B*, *C*, *D*, *E*, and *F* are undetermined coefficients, *x* is the abscissa of the center of the ellipse, and y is the ordinate of the center of the ellipse.

Using Equation (9) to process the discrete points after edge detection by least squares, each coefficient in the equation can be obtained. That is, each coefficient is determined by finding the minimum value of the objective Equation (10):

Through the extreme value principle, if you want the minimum value of $f^{\;}\left(A,B,C,D,E\right)$, then

(10) $$f^{\;}\left(A,B,C,D,E\right)={\left(\sum_{i=1}^{n}Ax_{i}^{2}+Bx_{i}y_{i}+Cy_{i}^{2}+Dx_{i}+Ey_{i}+F\right)}^{2}$$

Through the extreme value principle, if you want the minimum value of $f^{\;}\left(A,B,C,D,E\right)$, then

(11) $$\frac{\partial f}{\partial B}=\frac{\partial f}{\partial C}=\frac{\partial f}{\partial D}=\frac{\partial f}{\partial E}=\frac{\partial f}{\partial F}=0$$

Therefore, the linear equations are obtained through Equation (11). The values of equation coefficients *A*, *B*, *C*, *D*, *E*, and *F* can then be obtained by using the solution algorithm.

**Figure 4:**
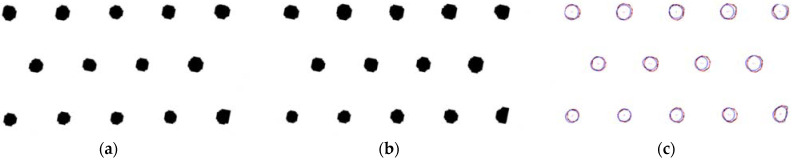
Contrast image of material surface profile and displacement: (**a**) before heating up; (**b**) after heating up; (**c**) Deformation results of vent hole.

In Figure 4a,b, the profiles of the circular array structure on the material surface before and after heating are collected respectively and compare the displacement of the contour structure before and after heating. The results are shown in Figure 4c. It can be found from Figure 4c that the displacement change of circular array structure is relatively small. The results show that the material is well protected at high temperature, and its circular array can maintain a stable structure.

## 3. Numerical Model and Result

### 3.1. Numerical Model

**Figure 5:**
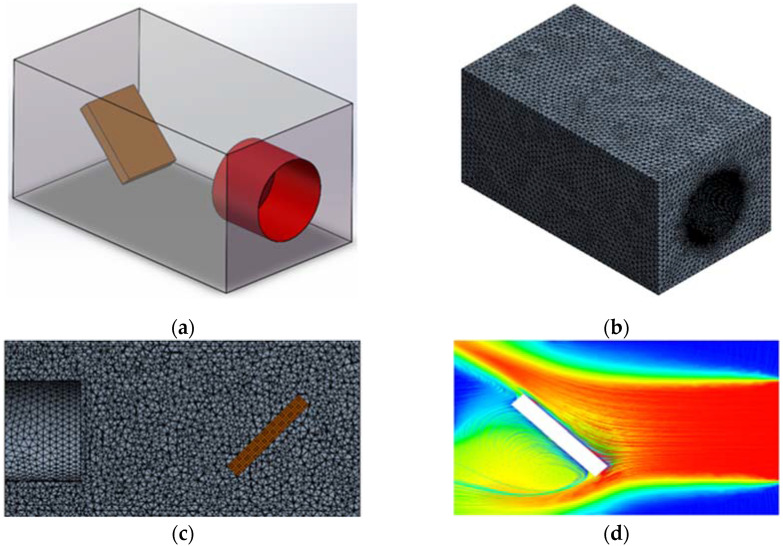
High temperature heating numerical model diagram: (**a**) model diagram; (**b**,**c**) grid model diagram, (**d**) high temperature airflow streamline diagram.

The main purpose of this study is to analyze the temperature field and deformation field of the material under high temperature environment. In order to reveal the behavior of the material at high temperature, a numerical model was established, as shown in Figure 5. The material in this model is tilted 45°, and the inlet of high temperature airflow is facing the material surface. In Figure 5a, the red surface is set as wall. The red wall directly facing the material is the velocity inlet. The velocity is 600 m/s, and the air flow temperature is about 1300 K. The overall outer surface of the model is set as the pressure outlet. A high quality structural grid is used for partition, as shown in Figure 5b,c. Figure 5d shows the distribution of high temperature air flow in the whole fluid domain.

**Figure 6:**
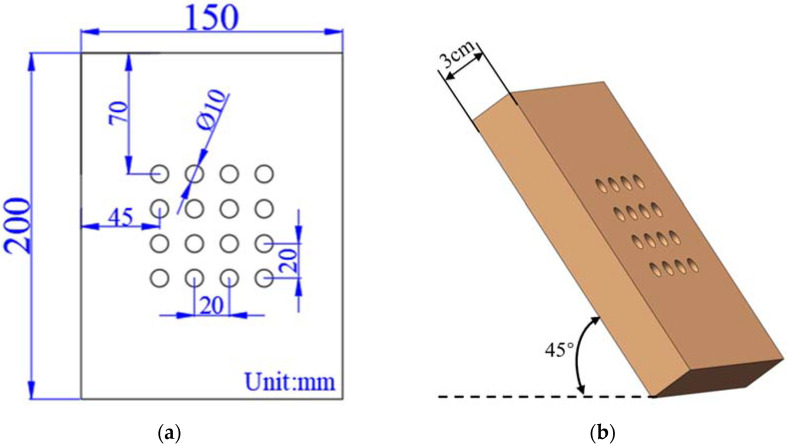

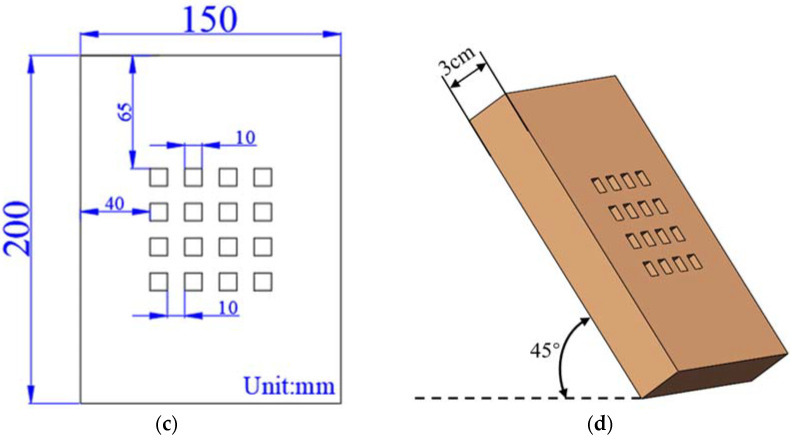
Schematic diagram of material size and location: (**a**) dimension of circular hole sample; (**b**) thickness and angle of circular hole sample; (**c**) dimension of square hole sample; (**d**) thickness and angle of square hole sample.

The size and location of the two material models are shown in Figure 6. The surface of the material is set as a round hole array and square hole array, respectively, and the overall size, thickness, and inclination angle of the material are given.

The two material parameters are listed in Table 1.

### 3.2. Results

**Figure 7:**
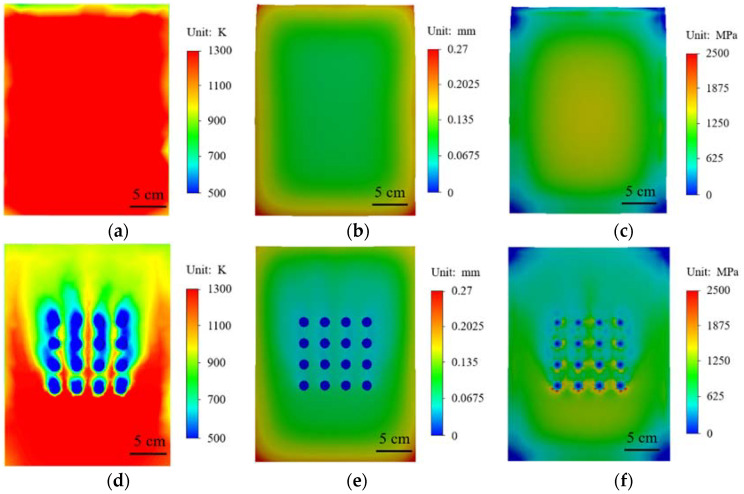
With or without circular structural material cloud diagram: (**a**–**c**) without prefabricated structural material; (**a**) temperature cloud diagram; (**b**) displacement cloud diagram; (**d**,**f**) prefabricated circular hole structure; (**c**) stress cloud diagram; (**d**) temperature cloud chart; (**e**) displacement cloud chart; (**f**) stress cloud chart.

In order to accurately understand the behavior changes of materials in high temperature environments, numerical simulation methods are used to conduct high temperature heating analysis of materials. High temperature heating simulation is carried out for the material with no structure on the surface and the material with a prefabricated circular hole array on the surface, and the results are shown in Figure 7. The surface temperature of the material without any structure is higher after heating, as shown in Figure 7a. Figure 7b shows the deformation field of the material after high temperature heating. The maximum deformation of the material after thermal expansion occurs at the corner of the surface edge. Figure 7c shows the stress distribution of the material after heating. Figure 7d shows that when there is 4 × 4 circular array structure on the surface of the material and airflow at normal temperature is discharged from the circular array structure, it can be seen that the temperature near the circular array will be greatly reduced after high temperature heating. The temperature above and below the circular array structure is in sharp contrast. It shows that circular array structure has a good cooling effect. In Figure 7e, near the circular array, the deformation of the material is significantly reduced. It can be seen from Figure 7f that the stress near the circular hole array is also significantly reduced, but due to the higher temperature, a higher stress distribution appears at the bottom row of circular hole structures.

**Figure 8:**
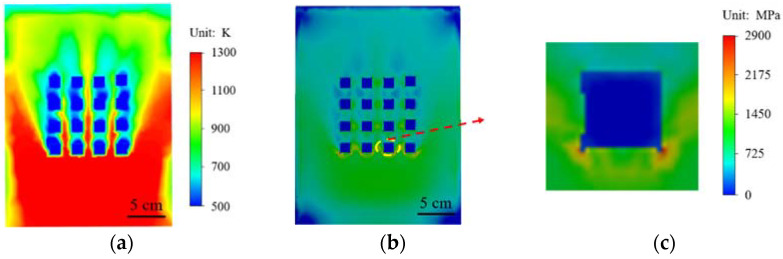

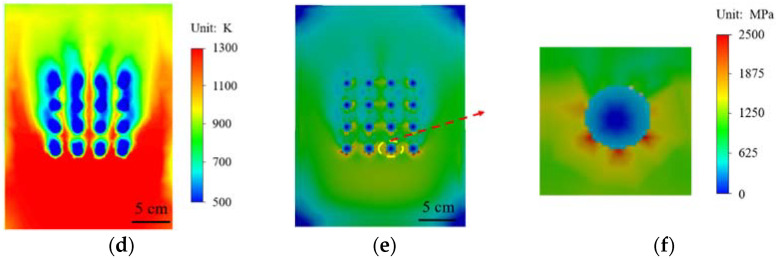
Comparison of square hole structure and round hole structure: (**a**–**c**) square structure; (**a**) temperature nephogram; (**b**) stress nephogram; (**c**) local enlarged stress nephogram; (**d**,**f**) circular structure; (**d**) temperature nephogram; (**e**) stress nephogram; (**f**) local enlarged stress nephogram.

The temperature field and stress state of square hole array and circular hole array under high temperature heating are compared in Figure 8. It can be seen from Figure 8a,d that the temperature nephogram of the square array structure is almost the same as that of the circular array structure. There is obvious cooling near and above the array structure. Figure 8b,e show the stress distribution of the two array structures in a high temperature environment, and there is almost no difference in the overall stress distribution of the material. However, in the enlarged drawings in Figure 8c,f of Figure 8b,e, it can be seen from the comparison that the square structure in Figure 8c appears to contain a stress concentration phenomenon at the corners, and in Figure 8f the stress distribution of the circular structure is around the circle. From the stress distribution of the two structures, the stress concentration phenomenon of the square structure is more likely to cause damage to the material structure.

**Figure 9:**
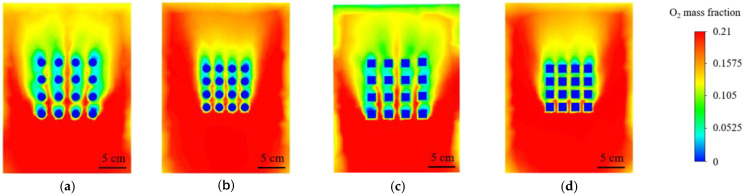
Oxygen concentration distribution diagram: (**a**) round hole structure; (**b**) small pitch round hole structure; (**c**) square hole structure; (**d**) small pitch square hole structure.

In a high temperature environment, the materials are easily oxidized, which will destroy the thermal protection performance of the material and increase the probability of material damage. In Figure 9, the oxygen concentration distribution of the circular array structure and the square array structure under high-temperature airflow is compared. In the two array structures shown in Figure 9b,d, the spacing of the array structure is reduced to half of the original. Comparing the oxygen concentration, it can be seen that the oxygen concentration distribution in the small spacing array model is larger than in the large spacing model. Therefore, it can be seen that the change of spacing have a significant effect on the surface oxygen concentration distribution. The decrease of oxygen concentration means that the oxidation area of the material is reduced, which will significantly improve the heat and oxidation resistance of the material.

**Figure 10:**
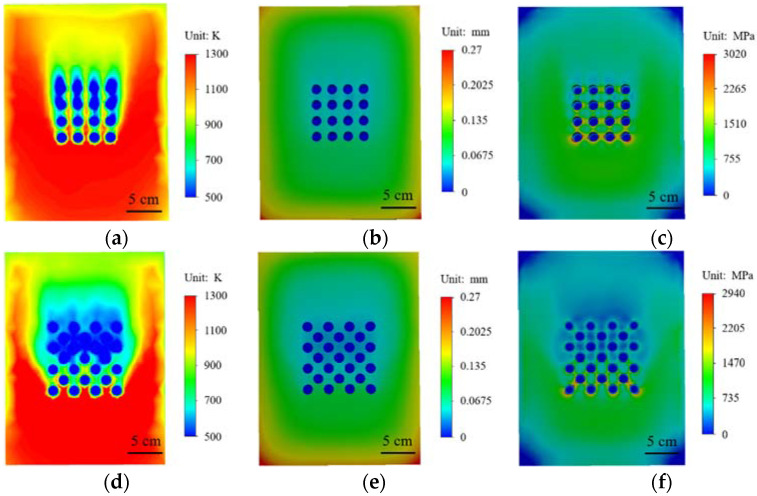
Small pitch round hole structure (**a**–**c**): (**a**) temperature nephogram; (**b**) stress nephogram; (**c**) local enlarged stress nephogram; Circular porous structure (**d**–**e**): (**d**) temperature nephogram; (**e**) stress nephogram; (**f**) local enlarged stress nephogram.

Comparing different surface structures, it can be found that compared with the square structure, the circular structure can better reduce the temperature and maintain the stability of the material structure. The circular structure can better meet the needs of high-temperature environments. The circular structure can better meet the needs of high-temperature environments. Two kinds of array structures of small spacing circular holes and circular porous structure were designed, and high-temperature heating simulations performed on them, as shown in Figure 10. It can be seen from Figure 10a that the small spacing circular array has a higher temperature in the gap compared to the circular porous array. For the surface temperature of the material, the cooling effect of the small spacing circular array structure is relatively small. Figure 10b,c shows the deformation field and stress field of small spacing circular holes. In Figure 10d–f, a circular porous structure is adopted. In Figure 10a, the circular array structure has an obvious cooling effect in high temperature environment, and the cooling effect at the array is more significant than other structures, indicating that the array structure can play a good role in thermal protection. In Figure 10e, the deformation of the material is also significantly reduced, especially near the array structure. The stress at the circular porous array structure is also relatively small, as shown in Figure 10f. Compared with different array structures, the circular porous array structure has a more excellent effect of cooling, reducing deformation and stress.

**Figure 11:**
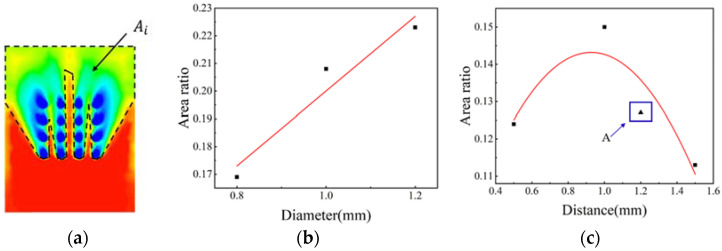
Area ratio of 500–1000 K surface temperature: (**a**) area selection diagram; (**b**) relationship between diameter of circular hole and area ratio; (**c**) relationship between spacing of circular hole and area ratio.

Comparing different structures, it can be concluded that the circular array structure has a better thermal protection effect. Therefore, the temperature distribution of the circular array structure with different diameter and spacing is compared. The temperature distribution state of 500–1000 K under different diameters and different spacing array structures on the material surface is collected as shown in Figure 11. Figure 11a shows the schematic diagram of surface temperature distribution collection. Figure 11b shows the area ratio of 500–1000 K temperature distribution under different diameters. It can be seen that as the diameter of the circular structure increases, the area distribution of 500–1000 K also becomes larger, indicating that the increase in diameter will increase the cooling effect to a certain extent. The temperature distribution area ratio of 500–1000 K under a circular array structure with different spacing is shown in Figure 11c. In order to verify the temperature change trend, the structure with a spacing of 1.2 cm is selected for verification analysis, marked point A as shown in Figure 11c. It can be seen that with the increase of the spacing, the area ratio of 500–1000 K temperature range decreases in a certain range. So, within a certain range, the larger the distance, the worse the cooling effect. The area ratio calculation formula is as follows:

(12) $$\beta =\frac{A_{i}}{S}$$

where *A_i_* represents the total area of 500–1000 K on the surface of the material at high temperature, *S* represents the total area of the material surface, and *β* represents the ratio.

**Figure 12:**
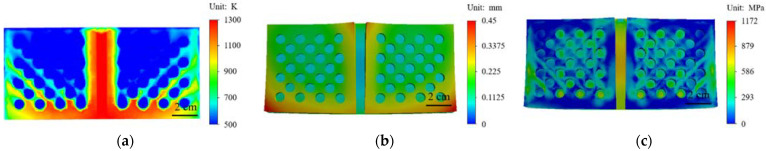
Horizontal splicing component nephogram: (**a**) temperature nephogram; (**b**) displacement nephogram; (**c**) stress nephogram.

Through the above comparison, it can be found that the cooling effect of the porous circular array structure is more obvious. Therefore, a transverse splicing structure is established on the basis of this structure. The splicing component base also uses the same material. The high-temperature heating simulation was performed on the structure, and the initial splicing gap width is 10 mm [3]. The temperature nephogram, deformation nephogram, and stress nephogram of the transverse splicing component are shown in Figure 12. It can be seen from the temperature cloud diagram in Figure 12a that the gaps of the transverse splicing members are more severely impacted by high temperature heat flow, the temperature in the gaps of the splicing parts is higher, and the surface temperature of the material is relatively low. The deformation nephogram after high temperature heating is shown in Figure 12b. The bottom gap is reduced due to the first contact of the heat flow at the bottom. Due to the impact of high temperature heat flow, the top of the gap presents with warping, which leads to the increase of the top gap. Figure 12c shows the stress distribution of the splicing piece, and the stress is more obvious at the gap.

**Figure 13:**
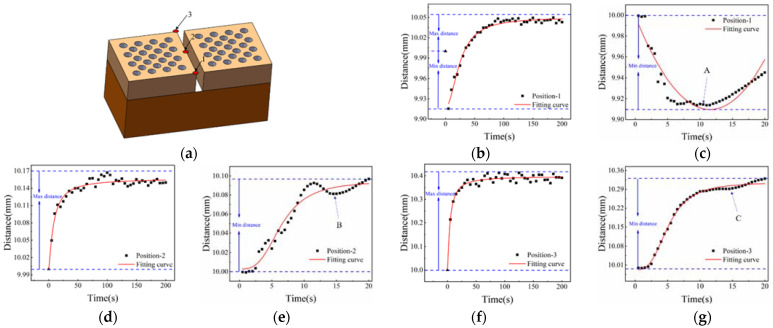
Gap change diagram of horizontal splicing component within 200 s: (**a**) horizontal stitching model diagram; (**b**) gap change at position-1; (**c**) Gap change at position-1 within 20 s; (**d**) Gap change at position-2; (**e**) gap change graph at position-2 within 20 s; (**f**) Gap change at position-3; (**g**) gap change graph at position-3 within 20 s.

In the transverse splicing component, three points are selected, and the gap changes of three positions are calculated, as shown in Figure 13a. The gap at position 1 in Figure 13b shows a trend that first decreases, then increases, and finally stabilizes. The gap spacing is reduced to about 9.91 mm. Figure 13c shows the trend of the gap change at position 1 within 20 s. The gap is subjected to thermal shock, and the expansion of the substrate causes the gap distance to decrease initially, and then increase. The minimum value appears at the mark A. Figure 13d,f record the gap changes of position 2 and 3. Compared with position 1, the gap width does not decrease, but gradually increases and then tends to be stable. The reason for this is that positions 2 and 3 are relatively backward, the time of contacting heat flux is relatively late, and the thermal expansion of the substrate is earlier than positions 2 and 3. In Figure 13e,g, the gap changes at positions 2 and 3 within 20 s are recorded, and the width of the gap increases gradually.

The marks B and C in Figure 13e,g indicate that in high temperature environments, the impact of high temperature heat flow on positions 2 and 3 gradually decreases.

**Figure 14:**
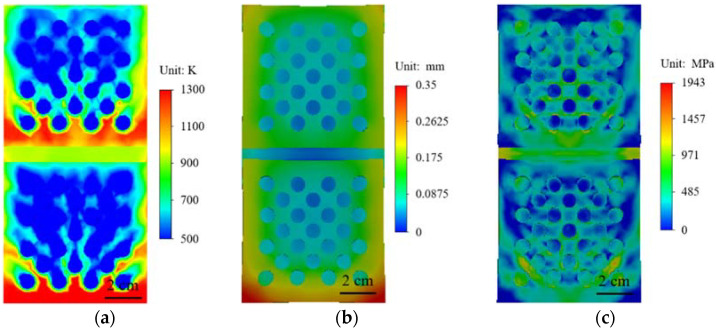
Longitudinal splicing component nephogram: (**a**) temperature nephogram; (**b**) displacement nephogram; (**c**) stress nephogram.

In order to more accurately understand the thermal expansion behavior of the splice, the gap width of the splice is 10 mm. The temperature nephogram, deformation nephogram, and stress nephogram of longitudinal splicing components are shown in Figure 14. As shown in Figure 14a, in the longitudinal splicing component, compared with the transverse splicing component, the gap temperature is significantly lower because the gap does not directly face the heat flow. It can be seen from the deformation nephogram in Figure 14b that the gap spacing decreases greatly at both ends and slightly in the middle. It can be seen from the deformation cloud in Figure 14b that the gap spacing decreases greatly at both ends and decreases slightly at the middle position. The maximum deformation occurs at the bottom of the splicing component. The stress distribution of longitudinal spliced components is shown in Figure 14c, and compared with the transverse splicing members, the stress at the gap decreases significantly.

**Figure 15:**
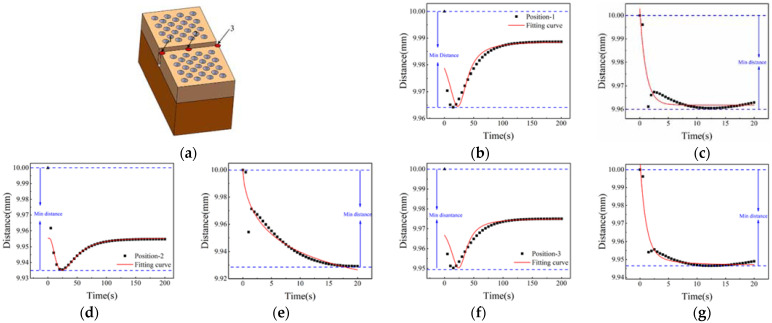
Gap change diagram of Longitudinal splicing component within 200 s: (**a**) horizontal stitching model diagram; (**b**) gap change at position-1; (**c**) Gap change at position-1 within 20 s; (**d**) Gap change at position-2; (**e**) gap change graph at position-2 within 20 s; (**f**) Gap change at position-3; (**g**) gap change graph at position-3 within 20 s.

Three positions are selected in the longitudinal splicing component gap, and the gap spacing changes are statistically analyzed In Figure 15. The schematic diagram of the material model and the distribution of the points as shown in Figure 15a. Figure 15b shows the gap change process at position 1. It can be seen that the gap spacing first decreases, then increases, and finally tends to be stable. The gap changes of position 1 within 20 s are shown in Figure 15c. Due to the influence of the side heat flow, the gap at position 1 shows a decreasing trend within 20 s. In Figure 15d,f the change process of the gap distance at positions 2 and 3 is recorded. Positions 2 and 3 also show a decrease first, then increase, and finally tend to be stable. Figure 15e,g show the gap change process of positions 2 and 3 within 20 s. The change of gap spacing shows a gradually decreasing trend, and the position 3 is affected by the side heat flow and has the same trend as position 1. In the longitudinal splicing components, the gap spacing generally decreases first, then increases, and finally tends to a stable state of change. In splicing components, when the spacing is reduced to a gradually stable state, the gap spacing of the splicing components can be optimized to achieve the optimal gap width of the splicing structure.

## 4. Conclusions

In this paper, high nitrogen steel samples with prefabricated pores are heated using a high temperature arc wind tunnel. The surface images of materials in high temperature environment are collected by temperature synchronous deformation measurement equipment, and the changes of temperature and pores are analyzed. Then, the numerical model is established through the numerical simulation method, and the heating simulation is carried out to further explore the high nitrogen steel material with prefabricated pores in the high-temperature environment. According to the simulation results, the prefabricated structure plays a key role in the high-temperature environment and has good thermal protection effect. The following conclusions can be drawn based on the experimental and numerical results:

(1) Through the temperature synchronous deformation measurement equipment, the image of the high nitrogen steel material with prefabricated pores under the high temperature arc wind tunnel is collected. The prefabricated structure and materials are well preserved under the high temperature arc wind tunnel, and the temperature is up to 1500 K, indicating that the prefabricated pore structure has a good thermal protection effect on the high nitrogen steel material.
(2) In the numerical simulation, different array structures are compared. The simulation results show that the porous circular array structure has a better thermal protection effect than other structures, and the stress concentration is smaller than other array structures, indicating that the porous array structure has a better thermal protection effect in high temperature environments.
(3) Finally, in the numerical simulation, the spliced components are established by using the porous array structure, the heating simulation is carried out in different directions of the spliced components, the temperature and stress nephogram of the spliced components are given, and the variation law of the gap of the spliced components is obtained, which provides theoretical and experimental support for the further application of material.

## Figures and Tables

**Table 1:** Material parameters for the numerical simulations.

Type	Density (kg/m^3^)	Specific Heat (J/(kg·K))	Thermal Conductivity (W/(m·K))	Coefficient of Thermal Expansion (K^−1^)
Cr18Mn18N	7540	460	30	0.6 × 10^−5^
Air	-	1006.43	0.0242	-

## Data Availability

Not applicable.
